# *Plasmodium* Parasite Malate-Quinone Oxidoreductase Functionally Complements a Yeast Deletion Mutant of Mitochondrial Malate Dehydrogenase

**DOI:** 10.1128/spectrum.00168-23

**Published:** 2023-04-10

**Authors:** Takeshi Ito, Sayaka Kajita, Minori Fujii, Yasuo Shinohara

**Affiliations:** a Institute of Advanced Medical Sciences, Tokushima University, Tokushima, Japan; b Graduate School of Pharmaceutical Sciences, Tokushima University, Tokushima, Japan; c Faculty of Pharmaceutical Sciences, Tokushima University, Tokushima, Japan; National Institutes of Health

**Keywords:** MQO, *Plasmodium*, energy metabolism, heterologous gene expression, mitochondria, yeast

## Abstract

The emergence of drug-resistant variants of malaria-causing *Plasmodium* parasites is a life-threatening problem worldwide. Investigation of the physiological function of individual parasite proteins is a prerequisite for a deeper understanding of the metabolic pathways required for parasite survival and therefore a requirement for the development of novel antimalarials. A *Plasmodium* membrane protein, malate-quinone oxidoreductase (MQO), is thought to contribute to the tricarboxylic acid (TCA) cycle and the electron transport chain (ETC) and is an antimalarial drug target. However, there is little information on its expression and function. Here, we investigated the function of Plasmodium falciparum MQO (PfMQO) in mitochondria using a yeast heterologous expression system. Using a yeast deletion mutant of mitochondrial malate dehydrogenase (MDH1), which is expected to be functionally similar to MQO, as a background strain, we successfully constructed PfMQO-expressing yeast. We confirmed that expression of PfMQO complemented the growth defect of the MDH1 deletion, indicating that PfMQO can adopt the metabolic role of MDH1 in energy transduction for growth in the recombinant yeast. Analysis of cell fractions confirmed that PfMQO was expressed and enriched in yeast mitochondria. By measuring MQO activity, we also confirmed that PfMQO expressed in yeast mitochondria was active. Measurement of oxygen consumption rates showed that mitochondrial respiration was driven by the TCA cycle through PfMQO. In addition, we found that MQO activity was enhanced when intact mitochondria were sonicated, indicating that the malate binding site of PfMQO is located facing the mitochondrial matrix.

**IMPORTANCE** We constructed a model organism to study the physiological role and function of P. falciparum malate-quinone oxidoreductase (PfMQO) in a yeast expression system. PfMQO is actively expressed in yeast mitochondria and functions in place of yeast mitochondrial malate dehydrogenase, which catalyzes the oxidation of malate to oxaloacetate in the TCA cycle. The catalytic site for the oxidation of malate in PfMQO, which is a membrane-bound protein, faces into the mitochondrial matrix, not the mitochondrial inner membrane space. Our findings clearly show that PfMQO is a TCA cycle enzyme and is coupled with the ETC via ubiquinone reduction.

## INTRODUCTION

Malaria is a major global life-threatening disease caused by five *Plasmodium* parasites that are transmitted by infected female mosquitoes. Of these five *Plasmodium* parasites, P. falciparum causes the most severe malaria. In 2021, it was estimated there were 247 million malaria cases and 619,000 deaths (1). Due to the emergence of drug-resistant parasites, an understanding of the metabolic pathways needed for the survival of *Plasmodium* parasites is crucial for the development of novel antimalarial compounds (2, 3).

In eukaryotic cells, mitochondria have important functions, such as energy transduction, maintenance of redox balance, and synthesis of metabolic precursors (4). For energy transduction, *Plasmodium* parasites mainly use either substrate-level or oxidative phosphorylation, depending on their life cycle stage. Thus, mitochondrial oxidative phosphorylation is essential for parasite development at the mosquito stage, whereas parasites at the asexual blood stage mainly depend on cytoplasmic glycolysis and lactate fermentation (5–8). However, the mitochondrial electron transport chain (ETC), from dihydroorotate dehydrogenase to complex III, is still essential at the blood stages, albeit for *de novo* pyrimidine biosynthesis rather than energy transduction (9). In addition, the requirement of a partially functional tricarboxylic acid (TCA) cycle at the blood stages has been suggested (10–12). In summary, both the TCA cycle and the ETC play key roles in parasite metabolism, but these roles are not restricted to energy transduction. To understand these processes in detail, investigation of each relevant enzyme function in the mitochondria or the cytoplasm is necessary.

Unlike many other organisms, including mammals, *Plasmodium* parasites do not possess mitochondrial malate dehydrogenase (MDH), which is a soluble enzyme of the TCA cycle that catalyzes the oxidation of malate to oxaloacetate coupled with the reduction of NAD^+^ to NADH, although the presence of a cytoplasmic MDH has been suggested (13–16). Instead, *Plasmodium* parasites encode malate-quinone oxidoreductase (MQO), which couples the oxidization of malate to oxaloacetate with reduction of ubiquinone (UQ) to ubiquinol (UQH_2_). MQO, which is found in some bacteria and apicomplexan parasites, is a membrane protein with no homology to MDH (16–19). Because of its catalytic properties, it has been assumed that *Plasmodium* parasites use MQO in place of MDH as a TCA cycle enzyme in the mitochondria, contributing to the ETC through the reduction of UQ (10, 16, 20–22). However, although a detailed biochemical characterization of *Plasmodium* MQO was recently published (23), there is insufficient evidence to verify this assumption. Nevertheless, a number of reports support the hypothesis. For example, tracer analyses of stable isotope-labeled metabolites demonstrated a fully functional TCA cycle in P. falciparum, including the pathway that converts fumarate to aspartate, through malate and oxaloacetate; furthermore, this was blocked by the inhibition of complex III, which reoxidizes UQH_2_ (10, 12). Other researchers have reported malate-dependent mitochondrial respiration and membrane potential generation in permeabilized *Plasmodium* cells, both of which were also inhibited by complex III inhibitors (20, 24). Nevertheless, these observations do not directly indicate the involvement of MQO. On the other hand, it is not clear whether MQO would catalyze the oxidation of malate to oxaloacetate in the mitochondrial matrix or within the mitochondrial inner membrane space, although this is a critical point in determining whether MQO can contribute to the TCA cycle. This underlines the importance of a detailed investigation of the expression and function of MQO in mitochondria.

Various strategies can be employed to determine protein function, including single gene deletion, use of specific inhibitors, or the creation of recombinant systems in a model organism (9, 25, 26). Heterologous expression systems in nonpathogenic yeast have been applied to studies on eukaryotic proteins (27), including mitochondrial enzymes from P. falciparum (28–30), because of the following advantages: posttranslational modifications, expression in the appropriate organelles, fast and scalable growth, and easy genomic manipulation. Baker’s yeast, Saccharomyces cerevisiae, is one of the simplest eukaryotic model organisms and possesses three types of MDH (mitochondrial MDH1, cytosolic MDH2, and peroxisomal MDH3) (31–33), but not MQO. Of the three types of MDH, mitochondrial MDH1 participates in the TCA cycle in the mitochondrial matrix (31). An interesting question is therefore whether MDH1 could be replaced by parasite MQO, despite the fact that they show no homology and use different electron acceptors. By addressing this question through a series of biochemical analyses of MQO-expressing yeast strains, this study demonstrates the expression and function of P. falciparum MQO (PfMQO) in mitochondria.

## RESULTS

### Construction of PfMQO-expressing yeast.

MQO and MDH both catalyze the reaction that converts malate to oxaloacetate, but they reduce different cofactors, i.e., UQ and NAD^+^, respectively. Of the three types of MDH in S. cerevisiae, MDH1 is localized to the mitochondrial matrix and contributes to the TCA cycle there. To examine whether MDH1 can be replaced by PfMQO, we made a deletion mutant of *Mdh1* as a background strain (Δ*Mdh1*) and constructed a PfMQO-expressing mutant (Δ*Mdh1*/*PfMqo*) in this background. To construct Δ*Mdh1*/*PfMqo*, a codon-optimized version of the PfMQO gene was inserted into a centromere vector, pRS314-YA2P, which included a promoter of the yeast mitochondrial ADP/ATP carrier 2 (AAC2) gene. The aim was for the *PfMqo* gene to be translated into native PfMQO without any modification of its amino acid sequence, conserving its own mitochondrial targeting signal, if present (see Materials and Methods).

### Comparison of growth phenotype of WT, Δ*Mdh1*, and Δ*Mdh1*/*PfMqo* yeast strains.

**FIG 1:**
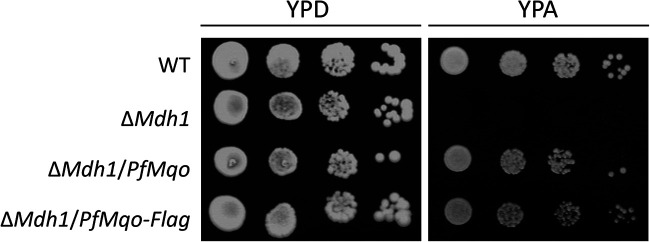
Growth phenotypes of yeast strains on fermentable and nonfermentable agar plates. Yeast cells (WT, Δ*Mdh1*, Δ*Mdh1*/*PfMqo*, Δ*Mdh1*/*PfMqo-Flag*) grown in SD selection medium were serially diluted, spotted on agar plates, and then incubated at 30°C for 3 days. YPD contains 2% glucose as a fermentable carbon source; YPA contains 2% acetate as a nonfermentable carbon source.

It has been reported that Δ*Mdh1* strains can grow on glucose, a fermentable carbon source, but not on acetate, a nonfermentable carbon source (32), where energy transduction through oxidative phosphorylation is required for cell growth; wild-type (WT) yeast can grow on both carbon sources. First, we confirmed that our Δ*Mdh1* strain showed the same growth phenotype as previously reported on these carbon sources (Fig. 1). Similarly, we tested the growth of Δ*Mdh1*/*PfMqo* and found that expression of PfMQO fully complemented the growth defect of Δ*Mdh1* on acetate plates (Fig. 1). This indicated that PfMQO is a functional substitute for MDH1, despite the fact that the two enzymes show no homology and use different electron acceptors.

### Localization of PfMQO expressed in yeast.

**FIG 2:**
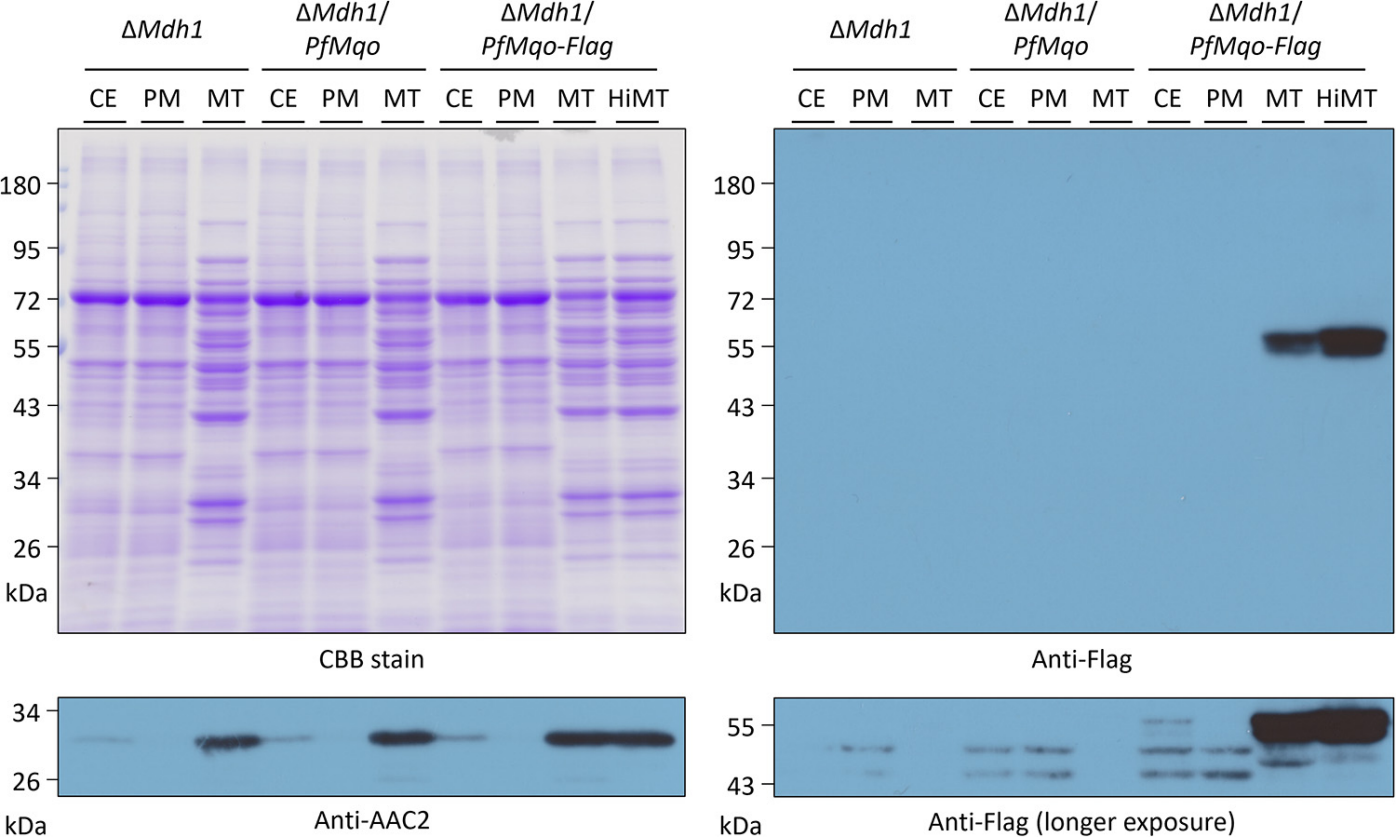
Western blot analysis showing the expression of PfMQO in yeast mitochondria. The Δ*Mdh1*/*PfMqo-Flag* yeast strain was grown in CMLE and fractionated into three fractions (CE, PM, and MT). The MT fraction of Δ*Mdh1*/*PfMqo-Flag* was further purified via sucrose gradient to obtain pure mitochondria (HiMT). Proteins (10 μg) from each fraction were subjected to 10% Laemmli SDS-PAGE and Western blotting. An antibody against the Flag tag at the C terminus of PfMQO was used for detection. As negative controls, fractions from Δ*Mdh1* and Δ*Mdh1*/*PfMqo* were used. Where indicated, an antibody against yeast inner mitochondrial membrane-bound ADP/ATP carrier 2 (AAC2) was used for a control experiment.

We next analyzed the localization and function of PfMQO in yeast cells. To verify whether PfMQO is expressed in yeast mitochondria, a mutant expressing PfMQO fused with a Flag tag peptide, Δ*Mdh1*/*PfMqo-Flag*, was constructed. We first confirmed that the growth defect of Δ*Mdh1* on acetate plates was complemented by the tagged version of PfMQO (Fig. 1). We then grew Δ*Mdh1*, Δ*Mdh1*/*PfMqo*, and Δ*Mdh1*/*PfMqo-Flag* in complete medium containing lactate and ethanol as nonfermentable carbon sources, in which Δ*Mdh1* was also able to grow, albeit slowly (34). We fractionated the cells into three types (cell extracts [CE], postmitochondrial fraction [PM], and mitochondrial fraction [MT]) and analyzed the fractions by Western blotting using an anti-Flag antibody. Δ*Mdh1* and Δ*Mdh1*/*PfMqo* were used as negative controls. An antibody against a peptide of yeast AAC2 (Ser2-Ser21) was used to detect the yeast inner mitochondrial membrane-bound ADP/ATP carrier 2, as a control experiment. AAC2 (34.4 kDa) was strongly visualized at ~30 kDa in the MT fraction and faintly in the CE fraction of each strain, but not in the PM fraction, which meant that cellular fractionation had been performed satisfactorily (Fig. 2). The estimated molecular weight of PfMQO with the Flag-tag was 60.5 kDa. Similarly to the control experiment with the AAC2 anti-peptide antibody, a strong band was observed around 60 kDa in the MT fraction of the Δ*Mdh1*/*PfMqo-Flag* strain, with a weaker band below, but not in the negative-control strains, indicating that the band represented PfMQO with the Flag tag (Fig. 2). The weaker band may have been a partially degraded product of PfMQO-Flag. The CE fraction of Δ*Mdh1*/*PfMqo-Flag*, which contained mitochondria, also showed a faint 60-kDa band after a longer exposure, although nonspecific bands appeared (Fig. 2). In contrast, there was no signal in the PM fraction, which suggested that PfMQO was specifically enriched in mitochondria. However, the MT fraction is unlikely to have comprised solely mitochondria and could contain several other cellular compartments. Thus, to confirm whether PfMQO is localized in mitochondria, the MT fraction of Δ*Mdh1*/*PfMqo-Flag* was further purified using a sucrose gradient, which allowed pure mitochondria (HiMT) to be isolated (35, 36). The HiMT of Δ*Mdh1*/*PfMqo-Flag* yeast showed stronger PfMQO-Flag bands than the less-pure MT fraction in Western blotting experiments (Fig. 2), which was consistent with the mitochondrial localization of PfMQO.

**FIG 3:**
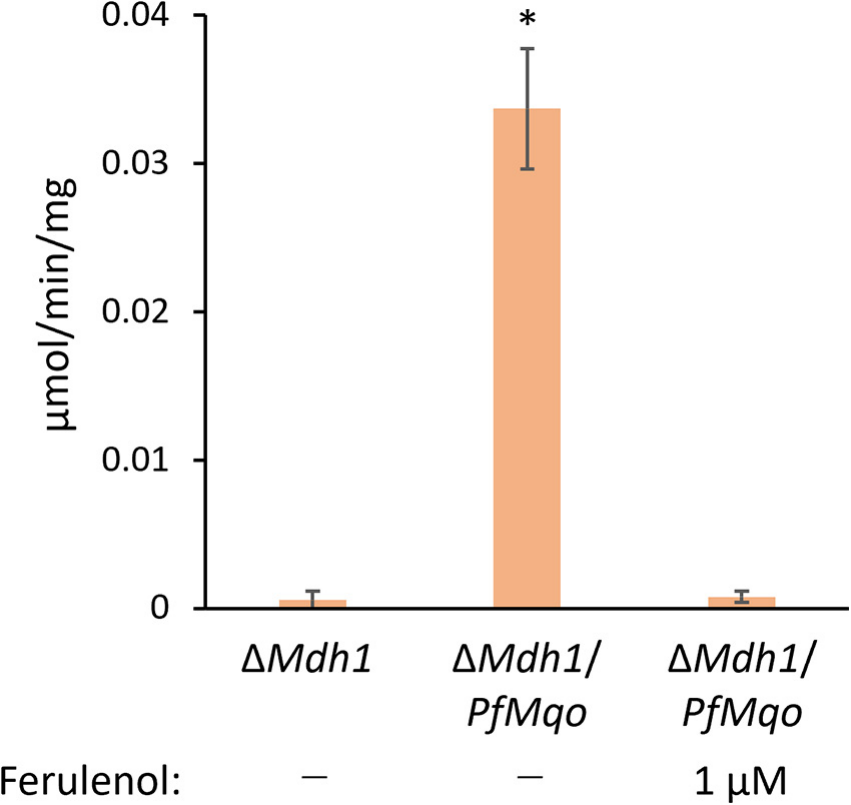
Measurements of PfMQO activity in yeast mitochondria. A mitochondrial fraction was isolated from Δ*Mdh1*/*PfMqo* yeast cells grown in CMLE medium. The malate-quinone oxidoreductase activity was measured using 60 μM decylubiquinone (dUQ) and 10 mM malate as substrates, and dUQ-dependent reduction of DCIP was monitored. Where indicated, 1 μM ferulenol was added as an MQO inhibitor. As a negative control, the mitochondrial fraction from Δ*Mdh1* was used. Measurements were repeated at least three times, and the error bars show standard deviations of the means. Data were analyzed by Dunnett’s test (*, *P* < 0.05), comparing Δ*Mdh1* and each other sample. EZR software was used (60).

Then, we examined whether PfMQO expressed in yeast mitochondria is active by measuring MQO activity. For activity measurements, the MT fractions of Δ*Mdh1* and Δ*Mdh1*/*PfMqo* were used. MQO activity was clearly present in the Δ*Mdh1*/*PfMqo* strain, but not in Δ*Mdh1*; moreover, the MQO activity in Δ*Mdh1*/*PfMqo* was almost completely suppressed by 1 μM ferulenol, a potent PfMQO inhibitor identified by Hartuti et al. (23) (Fig. 3). Altogether, our results clearly demonstrated that PfMQO is actively expressed in the recombinant yeast mitochondria.

### Respiratory activity of mitochondrial fractions.

To clarify whether PfMQO contributes to the TCA cycle and ETC, we measured respiratory activity driven by the TCA cycle using the mitochondrial fraction from Δ*Mdh1*/*PfMQO* cells grown in complete medium. WT and Δ*Mdh1* were used as a positive and negative controls, respectively. The respiratory activity assay was performed by measuring oxygen consumption rates with malate, pyruvate, and malate plus pyruvate as TCA cycle substrates. The TCA cycle requires the presence of both acetyl-coenzyme A (CoA) and oxaloacetate, which are produced from pyruvate and malate, respectively. Thus, when both malate and pyruvate are added (malate plus pyruvate), TCA cycle-dependent respiration is observed, and this activity is greater than the sum of the two individual activities (37). The efficiency factor of respiration coupled with the TCA cycle was determined as the ratio of the activity with the mixed substrates to the sum of the two individual activities (≈1.6 to 3.8 in S. cerevisiae [37–39]).

The results are shown in Table 1. The WT data showed that respiration coupled with the TCA cycle had a moderate efficiency ratio (≈2.3). Interestingly, deletion of *Mdh1* did not completely eliminate malate-dependent respiratory activity (with malate alone), while respiration coupled with a fully functional TCA cycle was effectively lost, according to the efficiency ratio (≈0.9). If the other yeast malate dehydrogenase(s), cytosolic MDH2 and/or peroxisomal MDH3, were able to contribute to the malate-dependent respiration observed, this would require the presence of a certain amount of either or both enzyme(s) together with NAD^+^ outside, but associated with, the mitochondria used in this assay. However, there was no significant contamination by malate dehydrogenases in the mitochondrial fraction of Δ*Mdh1* or Δ*Mdh1*/*PfMqo* at the enzyme activity level (see Fig. S1 in the supplemental material). Therefore, the results suggested that Δ*Mdh1* uses another pathway that enables malate-dependent respiration uncoupled from the TCA cycle. A candidate protein for this role is malic enzyme (MAE1), which is a mitochondrial enzyme that catalyzes the conversion of malate to pyruvate and the reduction of NAD^+^ to NADH. However, a double-deletion strain (Δ*Mdh1*/Δ*Mae1*) showed malate-dependent respiration with no significant difference from that of Δ*Mdh1* (44 ± 3 nmol O_2_/min/mg of protein), indicating that MAE1 is not involved in the malate-dependent respiration of Δ*Mdh1*. Another possibility is the conversion of malate to enol-oxaloacetate by mitochondrial succinate dehydrogenase, coupled with the reduction of quinone to quinol. The enol-oxaloacetate produced as a result works as a potent inhibitor of succinate dehydrogenase, but it can be converted to (keto-)oxaloacetate by aconitase (40). On the other hand, the expression of PfMQO restored the loss of TCA cycle-dependent respiration in Δ*Mdh1*, although the efficiency ratio was lower than in WT (about 1.5) (Table 1), which may reflect a difference in protein expression levels between PfMQO and MDH1 or a difference in their catalytic efficiencies under the experimental conditions used. Together, the results indicated that PfMQO can function as a TCA cycle enzyme by converting malate to oxaloacetate.

**TABLE 1:** TCA cycle-dependent respiratory rates of mitochondrial fractions^*a*^

Substrate(s)	Respiratory rate in strain		
	WT	Δ*Mdh1*	Δ*Mdh1*/*PfMqo*
Malate	27 ± 2	40 ± 3	37 ± 3
Pyruvate	10 ± 1	16 ± 3	11 ± 2
Malate + pyruvate (efficiency ratio)	84 ± 5 (2.3)	52 ± 3 (0.9)	73 ± 4 (1.5)

a Mitochondrial fractions were isolated from yeast cells (WT, Δ*Mdh1*, Δ*Mdh1*/*PfMqo*) grown in CMLE. Five millimolar malate, pyruvate, and malate plus pyruvate were used as respiratory substrates. Respiratory rates (in nanomoles of O_2_ per minute per milligram of protein) were measured in the presence of 250 μM ADP. The respiratory rate values represent means ± standard deviations. The efficiency ratio of respiration coupled with the TCA cycle was calculated by dividing the activity value in the presence of the mixed substrates by the sum of the individual activity values. Measurements were repeated three times.

**FIG 4:**
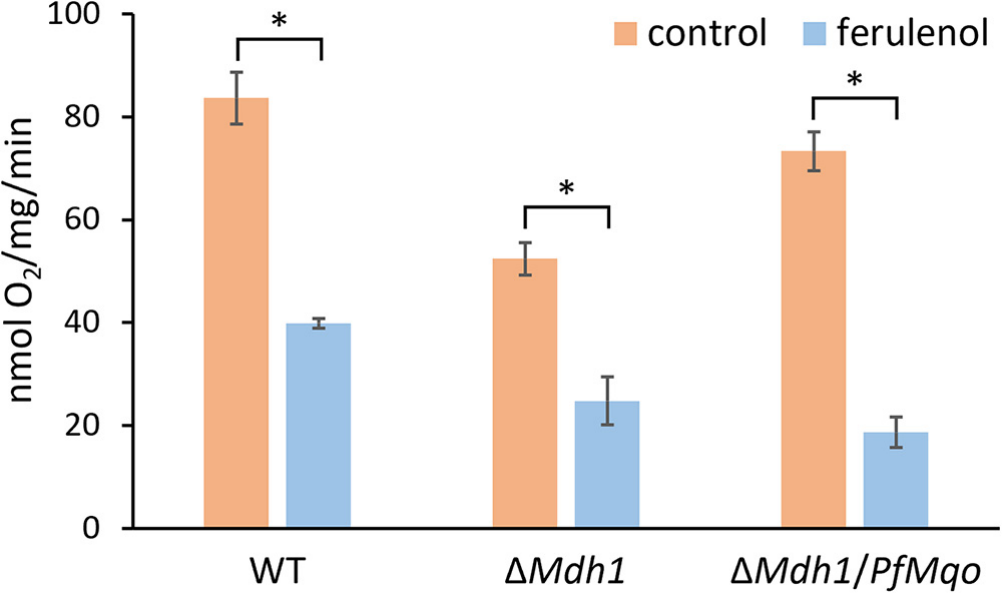
Inhibition of TCA cycle-dependent respiration with ferulenol. Mitochondrial fractions were isolated from yeast cells (WT, Δ*Mdh1*, Δ*Mdh1*/*PfMqo*) grown in CMLE. Five millimolar malate plus pyruvate was used as respiratory substrate. Respiratory rates were measured with 250 μM ADP in the absence or presence of 1 μM ferulenol. Measurements were repeated three times, and the error bars show standard deviations. Data were subjected to an unpaired *t* test (*, *P* < 0.05), comparing the results without ferulenol (control; shown in orange) and with 1 μM ferulenol (ferulenol; shown in blue).

We also measured the respiratory activity with malate plus pyruvate in the presence of the MQO inhibitor ferulenol. Ferulenol (1 μM) inhibited about 50% of the respiratory activity in WT and Δ*Mdh1* yeast (Fig. 4), which was consistent with our observation of its moderate inhibitory effect on yeast succinate dehydrogenase (50% inhibitory concentration [IC_50_] of ≈0.1 μM) (Fig. S2) and was not incompatible with the idea that the malate-dependent respiration in Δ*Mdh1* could derive from this enzyme. The respiratory activity in Δ*Mdh1*/*PfMqo* yeast was inhibited more strongly by 1 μM ferulenol (≈75%) (Fig. 4), because of the additional target (i.e., PfMQO) present in the strain, confirming that PfMQO contributes to both the energy metabolism of the TCA cycle and the ETC in yeast mitochondria. It is of interest that ferulenol is known to inhibit several ETC dehydrogenases, including MQO and succinate dehydrogenase, but the degree of inhibition in this class of enzyme differs among species (41). Similarly, the reported sensitivity to ferulenol in rat succinate dehydrogenase (IC_50_ of 17 μM and no apparent inhibition at 1.5 μM) was much weaker than our observation for yeast succinate dehydrogenase (42).

### Sidedness of PfMQO in mitochondria.

**FIG 5:**
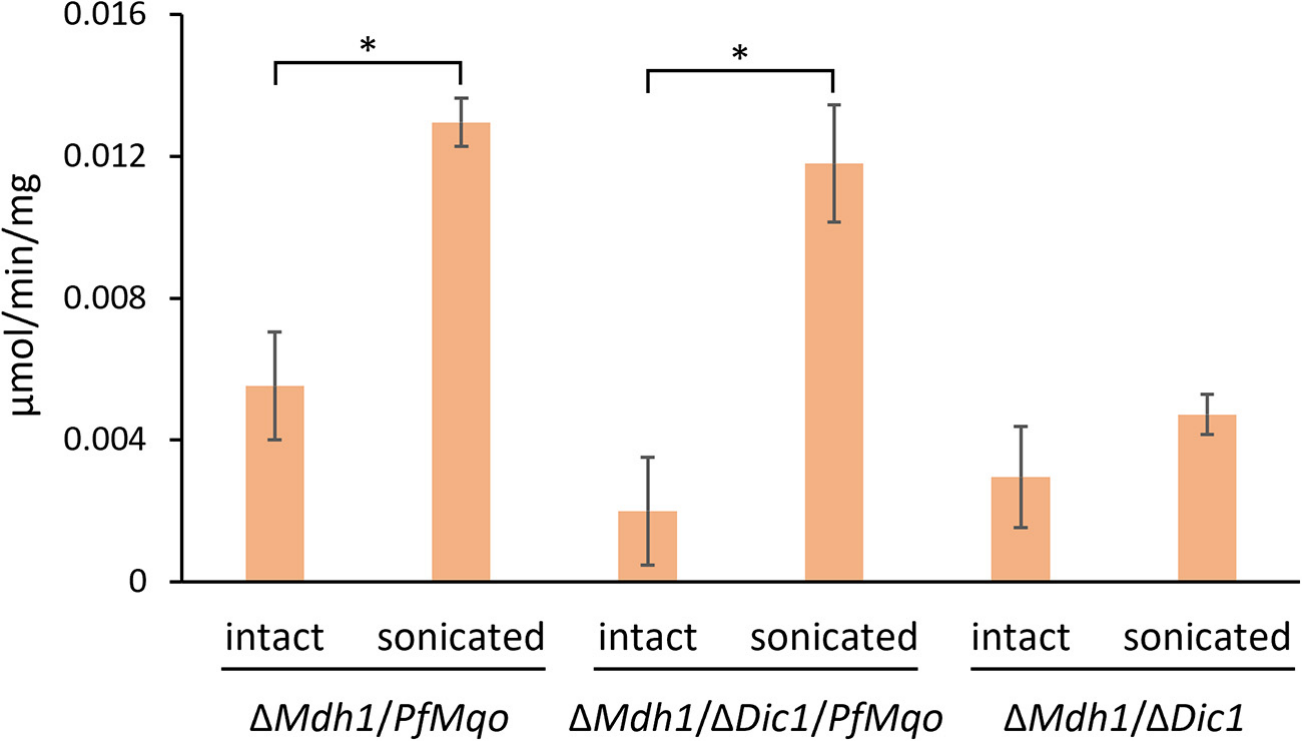
Sidedness analysis of the malate binding site of PfMQO. Mitochondrial fractions were isolated from yeast cells (Δ*Mdh1*/*PfMqo*, Δ*Mdh1*/Δ*Dic1*/*PfMqo*, Δ*Mdh1*/Δ*Dic1*) grown in CMLE. A portion of the fraction containing intact mitochondria was sonicated as appropriate. Malate-quinone oxidoreductase activity was measured using 60 μM dUQ and 10 mM malate as substrates. dUQ-dependent reduction of DCIP was monitored. As a negative control, the mitochondrial fraction from Δ*Mdh1*/Δ*Dic1* cells was used. Measurements were repeated three times, and error bars show standard deviations of the means. Data were analyzed using an unpaired *t* test (*, *P* < 0.05), comparing intact and sonicated mitochondria.

To determine that the above functions of PfMQO take place in the appropriate organellar subcompartment, we needed to examine whether the malate binding site in MQO was directed toward the mitochondrial matrix. There is a report showing that the oxidation of malate to oxaloacetate within a fully functional TCA cycle does not necessarily occur in the mitochondrial matrix in yeast cells (43), possibly because of the presence of malate and oxaloacetate carriers in the mitochondrial inner membrane. To investigate the sidedness of PfMQO in mitochondria, we measured the MQO activity using intact or sonicated mitochondria from the Δ*Mdh1*/*PfMQO* yeast strain grown in complete medium. If the malate-binding site is localized in the mitochondrial matrix, membrane-impermeable malate, added externally, must be transported into the matrix via inner mitochondrial membrane-bound carriers, such as the dicarboxylate carrier (DIC1), which could be a rate-limiting step for the MQO reaction in intact mitochondria but not in sonicated organelles. The results showed that the breakage of mitochondrial membranes by sonication significantly enhanced the activity of MQO (Fig. 5). To rule out the possibility that this observation was due to the improved accessibility of the other substrate, i.e., decylubiquinol and/or DCIP, rather than of malate, we next examined a deletion strain of *Dic1*, which encodes the main carrier for the uptake of malate into the matrix. The Δ*Mdh1*/Δ*Dic1*/*PfMQO* yeast strain showed an even stronger effect of sonication than the Δ*Mdh1*/*PfMQO* strain (5.9- and 2.3-fold, respectively) (Fig. 5). This is presumably because the intact mitochondria in the *Dic1* deletion strain have a reduced ability to take up malate into the matrix, indicating that the malate binding site in MQO faces into the mitochondrial matrix. On the other hand, the Δ*Mdh1*/Δ*Dic1* double mutant showed no difference between intact and sonicated mitochondria (Fig. 5). It should be noted that the outer mitochondrial membrane, unlike the inner mitochondrial membrane, is highly permeable to molecules smaller than 5,000 Da, because of voltage-dependent anion channels in the outer mitochondrial membrane (44).

## DISCUSSION

**FIG 6:**
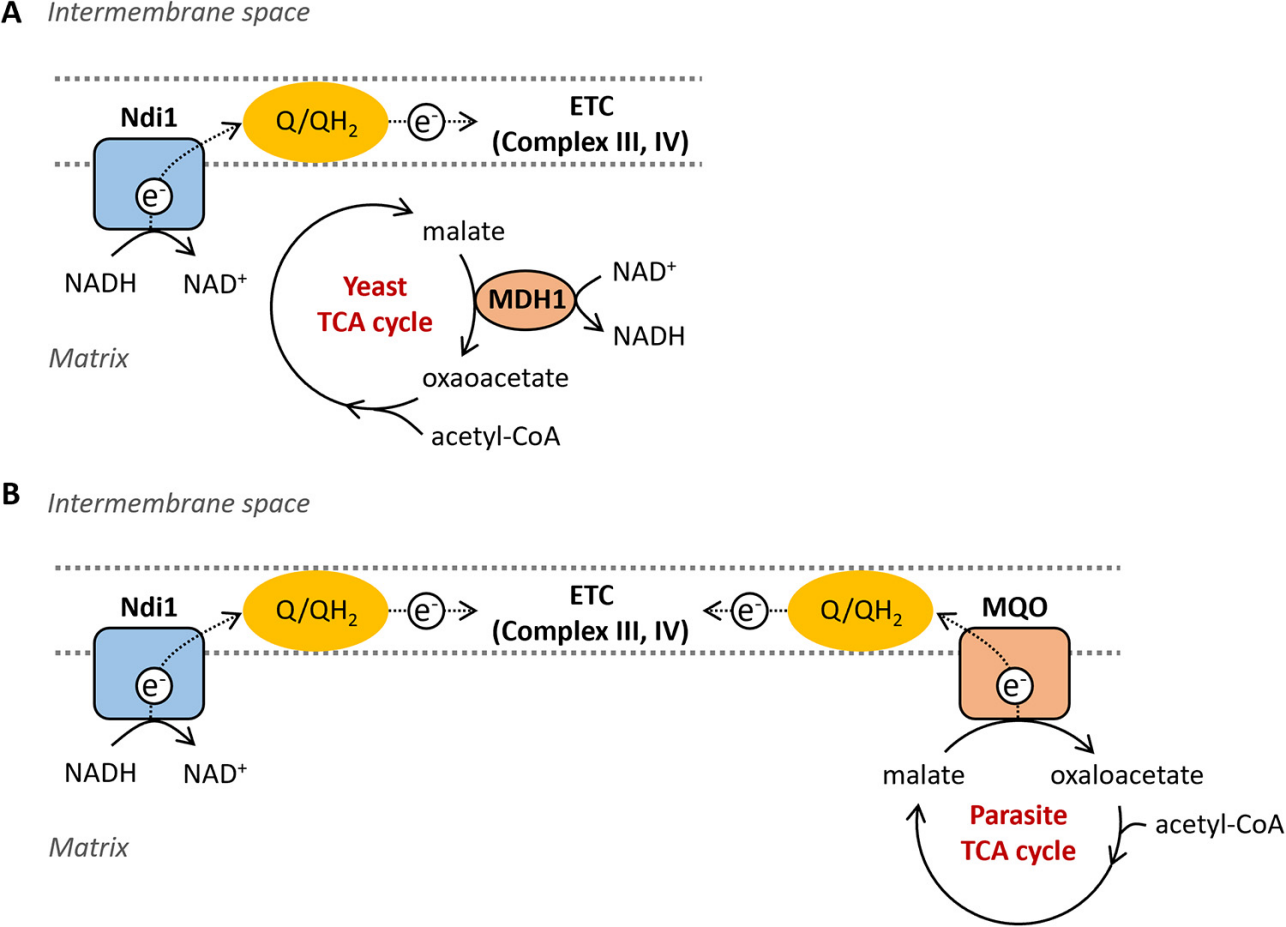
Mitochondrial TCA cycle and ETC in the yeast wild-type and parasite-type strain (Δ*Mdh1*/*PfMqo*). (A) In the yeast WT TCA cycle, mitochondrial malate dehydrogenase (MDH1) oxidizes malate to oxaloacetate and reduces NAD^+^ to NADH. Internal NADH dehydrogenase (Ndi1) oxidizes the NADH produced, and the resultant electrons are transferred to complex III through the quinone/quinol pool (Q/QH_2_) and then to complex IV. (B) In the parasite-type TCA cycle, malate-quinone oxidoreductase (MQO) catalyzes the conversion of malate to oxaloacetate coupled with the reduction of quinone to quinol, and then the electrons are transferred to complex III and complex IV. The NADH produced in mitochondria is also used through Ndi1.

In this study, we characterized the role of malate-quinone oxidoreductase from the malaria parasite P. falciparum (PfMQO). We found that yeast malate dehydrogenase MDH1 is functionally replaceable with PfMQO (Fig. 6). Using as a model organism a PfMQO-expressing yeast strain lacking MDH1 enabled us to clearly reveal the expression and enrichment of active PfMQO in mitochondria using Western blot analysis and measurements of MQO activity. The contribution of PfMQO to the energy metabolism of the TCA cycle and the ETC was then elucidated by a series of respiratory activity measurements and by analyses of the sidedness of its catalytic site for malate oxidation using mitochondria from our model organism.

To the best of our knowledge, this is the first report that unequivocally demonstrates the expression and function of *Plasmodium* MQO in mitochondria. Some reports have suggested a role for MQO in parasite metabolism, such as the TCA cycle, ETC, and the fumarate cycle serving the purine salvage pathway, based on metabolite analyses (10, 12, 45) or on respiratory activity measurements (20, 24). However, there was insufficient evidence that the results obtained were due to MQO and not to other proteins. To identify the function of MQO in mitochondria, experiments using its single-deletion mutant, a specific inhibitor, or a recombinant system with a model organism would be required. It must be noted that MQO activity was recently observed in a mitochondria-rich fraction of P. falciparum by measuring the malate-quinone-dependent reduction of DCIP (46), which suggested the expression of MQO in parasite mitochondria, but its contribution to the TCA cycle or mitochondrial respiration is still not clear. Also, in another apicomplexan parasite, Toxoplasma gondii, both MDH and MQO have been shown to be localized in mitochondria (47). However, there is no report on whether the malate binding site of MQO is directed toward the mitochondrial matrix and thus whether both contribute to TCA cycle function.

During the *Plasmodium* parasite life cycle, a fully functional TCA cycle is not essential except at the mosquito stage, although some TCA cycle reactions are required at the blood stages, probably for producing NADH to maintain NADH/NAD^+^ redox balance, preventing the accumulation of citrate to a potentially toxic level, and driving the fumarate cycle for the purine salvage pathway, rather than for energy transduction (10, 12). MQO and fumarate hydratase (FH) have been suggested as essential TCA cycle enzymes, coupled with the fumarate cycle serving the purine salvage pathway, for growth at the asexual blood stage, because these enzymes could not be genetically disrupted at this stage in the P. falciparum D10 strain despite multiple trials with a variety of approaches (10, 12). In addition, although these enzymes were not essential in a rodent malaria parasite, the Plasmodium berghei ANKA strain, the importance of MQO for growth and the requirement for MQO for full virulence at the asexual blood stage was confirmed (48). As a consequence, MQO has attracted attention as a promising drug target (22, 23, 49, 50). However, recently Rajaram et al. successfully disrupted the genes for MQO and FH in the P. falciparum NF54^attB^ strain and showed that these enzymes are nonessential for survival at the blood stage, based on a growth test of the double-knockout mutant (45). These contradictory observations in P. falciparum strains (D10 and NF54^attB^) indicate that different laboratory strains of the parasite may have varied dependencies on MQO and FH. This idea is supported by a report showing that reliance on mitochondrial ETC function is variable among P. falciparum strains (51). The present study supports the notion that MQO could be a novel antimalarial target at those life cycle stages where the TCA cycle or the conversion of malate to oxaloacetate in mitochondria is required. However, it is still unclear why the parasites use quinone-reducing MQO, but not NADH-producing mitochondrial MDH, like many other organisms. Also, while the biochemical properties of MQO from P. falciparum and T. gondii have been studied in detail (23, 52), no crystal structure is yet available, even for bacterial versions of the enzyme. In the latest report by Inaoka’s group, bacterial MQO from Campylobacter jejuni was successfully purified with very high specific activity; this represents important progress toward solving the structure (53). To understand the details of the role of MQO in parasite metabolism, however, further investigations will be required.

## MATERIALS AND METHODS

### Yeast strains and growth conditions.

S. cerevisiae strains were grown on media supplemented with the following carbon sources: 2% glucose, potassium acetate, or sodium lactate and ethanol. Yeast extract peptone medium (1% yeast extract; Nacalai Tesque, 2% hipolypepton N [Fujifilm Wako Pure Chemical Corporation]) was supplemented with glucose (YPD) or potassium acetate (YPA). Complete medium (1% yeast extract; Nacalai Tesque, 0.1% potassium phosphate, 0.12% ammonium sulfate; pH 5.5) was supplemented with sodium lactate (pH 5.5) and ethanol (CMLE). Synthetic minimal medium (0.67% yeast nitrogen base without amino acids [BD Difco]) was supplemented with glucose, all essential amino acids, and other nutrients to meet the auxotrophic requirements (SD). Plates contained 2% agar. When necessary, 0.2 mg/mL Geneticin (G418 sulfate) was added. Cells were grown at 30°C. Escherichia coli strains were grown in Luria-Bertani medium at 37°C. When necessary, 80 μg/mL ampicillin was added.

### Deletion of the *Mdh1*, *Mae1* and *Dic1* genes.

Disruption of *Mdh1* was performed in S. cerevisiae W303-1B (WT) by the one-step gene replacement method (54). The *Mdh1* deletion cassette containing a G418 sulfate resistance gene (*kanMX*) as a selection marker was amplified by PCR using pUG6 plasmid and the primers shown in Table S1. For selection of Δ*Mdh1* transformants, YPD agar plates supplemented with 0.2 mg/mL G418 sulfate were used. The *Mae1* and *Dic1* gene disruptions were constructed in the Δ*Mdh1* strain using the same method as for the deletion of *Mdh1*. The *Mae1* deletion cassette containing a histidine synthesis gene (*HIS3*) was amplified using pRS313 plasmid and the relevant primers (Table S1). For selection of Δ*Mdh1*/Δ*Mae1* transformants, SD agar plates lacking histidine, supplemented with 0.2 mg/mL G418 sulfate, were used. The *Dic1* deletion cassette containing a leucine synthesis gene (*LEU2*) was amplified using pRS305 plasmid and the relevant primers (Table S1). For selection of Δ*Mdh1*/Δ*Dic1* transformants, SD agar plates lacking leucine, supplemented with 0.2 mg/mL G418 sulfate, were used. Gene deletions were verified by PCR using external primers homologous to the flanking regions of the *Mdh1*, *Mae1*, or *Dic1* genes (Table S1 and Fig. S3).

### Construction of plasmids.

A codon-optimized cDNA of PfMQO (PF3D7_0616800), containing NdeI and BamHI restriction sites at the 5′ and 3′ ends, was synthesized and inserted into pUC57 at the NdeI and BamHI restriction sites (GenScript). The codon-optimized PfMQO cDNA was subcloned into pRS314-YA2P, which is a centromere vector containing a promoter of the yeast mitochondrial ADP/ATP carrier 2 (AAC2) gene, as described previously (55), to construct a PfMQO-expression plasmid (pRS314-YA2P/*PfMqo*). The aim of the design was for the *PfMqo* gene to be translated into the native PfMQO protein without any modification of its amino acid sequence, conserving its own mitochondrial targeting signal, if present. To add the Flag tag sequence to the C terminus of PfMQO, a region including the *PfMqo* gene with a Flag sequence, stop codon, and a BamHI restriction site at the 3′ end was amplified using pRS314-YA2P/*PfMqo* and the primers listed in Table S1. Then, the PCR product was digested with NdeI and BamHI and inserted into pRS314-YA2P to construct the C-terminally Flag-tagged PfMQO expression plasmid (pRS314-YA2P/*PfMqo-Flag*).

### Yeast transformation.

The deletion strains Δ*Mdh1* and Δ*Mdh1*/Δ*Dic1* were transformed by the lithium acetate method (56) with plasmids expressing PfMQO (pRS314-YA2P/*PfMqo*) or C-terminal Flag-tagged PfMQO (pRS314-YA2P/*PfMqo-Flag*). To select transformants, SD agar plates lacking tryptophan or lacking leucine and tryptophan, supplemented with 0.2 mg/mL G418 sulfate, were used.

### Growth tests.

Individual strains were grown in SD medium lacking tryptophan or tryptophan and with histidine as necessary. Then, 10-fold serial dilutions of cultures that had reached an optical density at 600 nm of 1, 0.1, 0.01, or 0.001 were spotted on YPD and YPA agar plates. Yeast can grow fermentatively on glucose but requires mitochondrial oxidative phosphorylation on nonfermentable carbon sources, such as acetate, ethanol, and lactate. It has been reported that S. cerevisiae strains lacking *Mdh1* grow slowly on nonfermentable media containing ethanol or lactate but have a significant growth defect on acetate medium (32, 34). Thus, only S. cerevisiae W303-1B (WT) and mutant strains in which the deletion of *Mdh1* is complemented can grow on acetate medium. Plates were incubated at 30°C for 3 days.

### Isolation of yeast mitochondria.

A mitochondrial fraction was isolated from yeast cells according to a method described by Luttik et al. (57). Yeast cells were grown in CMLE (containing lactate and ethanol as nonfermentable carbon sources). Cells were harvested by centrifugation (3,000 × *g*, 4 min) and washed with water. Cell pellets were then resuspended in 0.1 M Tris-H_2_SO_4_ (pH 8), 10 mM dithiothreitol (2 mL per g wet cell weight) and incubated at 30°C for 15 min with shaking at 70 rpm. After centrifugation (3,000 × *g*, 4 min), cell pellets were washed with buffer 1 (20 mM KP_i_ [pH 7.4], 1.2 M d-sorbitol). Cell pellets were then resuspended in the same buffer (6.7 mL per g wet cell weight), and zymolyase-T20 (10 mg per g wet cell weight; Nacalai Tesque) was added, after which cell suspensions were incubated at 30°C for 45 min with shaking at 70 rpm to prepare spheroplasts. Spheroplasts were harvested by centrifugation (2,200 × *g*, 7 min, 4°C) and washed with buffer 1. Spheroplast pellets were then resuspended in buffer 2 (10 mM Tris-HCl [pH 7.4], 0.6 M d-mannitol, 2 mM EDTA, 1 mg/mL fatty acid-free bovine serum albumin [6.7 mL per g wet cell weight]) and lysed in a tight-fitting Dounce homogenizer (10 strokes). Homogenates were separated from unbroken cells and debris by centrifugation (2,000 × *g*, 10 min, 4°C) to obtain cell extracts (supernatants).

The cell extracts were subjected to an additional centrifugation (7,800 × *g*, 10 min, 4°C) to separate two fractions, a mitochondrial fraction (pellet) and a postmitochondrial fraction (supernatant), and the resulting pellets, containing mitochondria, were resuspended in buffer 2 (50 μL per g wet cell weight). The cell extracts and the postmitochondrial fraction were stored at −80°C until use. The mitochondrial fraction was kept on ice for respiration measurements and sidedness analyses, or otherwise stored at −80°C until used alongside the other fractions. When necessary, the mitochondrial fraction was further purified via a sucrose gradient to exclude other cellular compartments, based on the method described by Meisinger et al. (35) and Gregg et al. (36). The mitochondrial fraction pellet was resuspended in SEM (3 mL) (10 mM MOPS-KOH [pH 7.2], 250 mM sucrose, 1 mM EDTA). This was overlaid on a step gradient of sucrose prepared in EM (10 mM MOPS-KOH [pH 7.2], 1 mM EDTA) containing (from the bottom upwards) sucrose at a concentration of 60% (1.5 mL), 32% (4 mL), 23% (1.5 mL), and 15% (1.5 mL), and was then subjected to ultracentrifugation (134,000 × *g*, 1 h, 4°C) using a swing-rotor P40ST (himac). A brown band between the 60% and 32% sucrose layers was carefully collected and pelleted by centrifugation (10,000 × *g*, 30 min, 4°C). The pellet of pure mitochondria obtained was resuspended in buffer 2 (25 μL per g wet cell weight) and stored at −80°C until use.

### Western blotting of Flag-tagged proteins.

Cell extracts, together with postmitochondrial and mitochondrial fractions, were prepared from Δ*Mdh1*, Δ*Mdh1*/*PfMqo*, and Δ*Mdh1*/*PfMqo-Flag* strains grown in CMLE. The mitochondrial fraction of Δ*Mdh1*/*PfMqo-Flag* was further purified by separation via sucrose gradient to obtain pure mitochondria. Samples containing 10 μg protein were subjected to 10% Laemmli SDS-PAGE. Separated proteins were then transferred to a polyvinylidene difluoride (PVDF) membrane by the glycine-methanol transfer system. The PVDF membrane was probed with a primary anti-Flag antibody from rabbit (0.2 μg/mL; Sigma-Aldrich) followed by an anti-rabbit IgG secondary antibody conjugated with horseradish peroxidase (Cytiva) for detection. For a control experiment, a primary antibody from rabbit against a peptide of yeast AAC2 (Ser2-Ser21) was used. The antibody was previously prepared to detect yeast inner mitochondrial membrane-bound ADP/ATP carrier 2 (58).

### Malate-quinone oxidoreductase activity.

Mitochondrial fractions were isolated from Δ*Mdh1* and Δ*Mdh1*/*PfMqo* strains grown in CMLE. The malate-dependent ubiquinone reduction in isolated mitochondrial fractions was measured spectrophotometrically based on the method described by Hartuti et al. (23). Malate-dependent ubiquinone reductase activity was measured spectrophotometrically at 37°C in a quartz cuvette. DCIP was used as an indicator to detect the production of ubiquinol. The absorbance change at 600 nm (ε = 21 mM^−1 ^cm^−1^) was monitored, which showed the reduction of DCIP by the ubiquinol produced. Mitochondrial fractions (0.05 mg/mL) and 120 μM DCIP (Sigma-Aldrich) were added to the assay buffer (1.8 mL) containing 50 mM HEPES-KOH (pH 7), 2 μM antimycin A, and were incubated for 1 min. Then, 60 μM decylubiquinone (dUQ [Enzo Life Sciences]) was added. After an additional incubation for 1 min, the reaction was initiated by adding 10 mM l-malate. Where appropriate, 1 μM ferulenol (Adipogen Life Sciences) was added to the assay buffer. Activity measurements were repeated at least three times.

### Respiration activity.

Mitochondrial fractions were isolated from S. cerevisiae W303-1B (WT) and the Δ*Mdh1*, Δ*Mdh1*/Δ*Mae1*, and Δ*Mdh1*/*PfMqo* strains, all of which were grown in CMLE. TCA cycle-dependent oxygen consumption rates were measured polarographically at 25°C with a Clark-type oxygen electrode (57). An aliquot of each mitochondrial fraction (0.14 mg/mL) was added to an air-saturated assay buffer (2.2 mL) containing 25 mM KP_i_ (pH 7), 0.65 M d-sorbitol, 5 mM MgCl_2_. The reactions were initiated by adding 5 mM l-malate, 5 mM pyruvate, or 5 mM l-malate plus 5 mM pyruvate as respiration substrates, and then 0.25 mM ADP was added. Substrate-dependent respiration rates in the presence of ADP were calculated by subtracting nonspecific mitochondrial oxygen consumption, which was determined under conditions without substrates. The oxygen concentration of the air-saturated assay buffer at 25°C was assumed to be 258.2 nmol O_2_/mL. All activity measurements were repeated at least three times.

### Sidedness of the malate binding site of MQO.

Mitochondrial fractions were isolated from S. cerevisiae Δ*Mdh1*/*PfMqo*, Δ*Mdh1*/Δ*Dic1*/*PfMqo*, and Δ*Mdh1*/Δ*Dic1* strains grown in CMLE. Where appropriate, part of the mitochondrial fraction (containing intact mitochondria) was sonicated constantly for 20 s on ice with a Branson sonifier 450 (59). The malate-dependent ubiquinone reduction in the intact or sonicated mitochondria was measured spectrophotometrically as described above with some modifications. Malate-dependent ubiquinone reductase activity was measured spectrophotometrically at 25°C using a quartz cuvette. DCIP was used as an indicator to detect the production of ubiquinol. The absorbance change at 600 nm (ε = 21 mM^−1 ^cm^−1^) was monitored, which showed the reduction of DCIP by the ubiquinol produced. Mitochondria (0.05 mg/mL) and 120 μM DCIP (Sigma-Aldrich) were added to the assay buffer (0.9 mL) containing 50 mM HEPES-KOH, 0.65 M d-sorbitol, 1 mM KP_i_ (pH 7), 2 μM antimycin A and then incubated for 1 min, after which 60 μM dUQ was added. Following an additional incubation for 1 min, the reaction was initiated by adding 10 mM l-malate. Activity measurements were repeated at least three times.

### Protein concentrations.

Protein concentrations were determined by the bicinchoninic acid method with bovine serum albumin solutions as standards.
