# Supplementary material for: Plasmodium Parasite Malate-Quinone Oxidoreductase Functionally Complements a Yeast Deletion Mutant of Mitochondrial Malate Dehydrogenase
Source: Microbiol Spectr. 2023 Apr 10;11(3):e00168-23. doi: 10.1128/spectrum.00168-23 (PMC10269487; doi:10.1128/spectrum.00168-23)
Supplement: Supplemental file 1 — Supplemental material. Download spectrum.00168-23-s0001.pdf, PDF file, 1.1 MB [file spectrum.00168-23-s0001.pdf]

**Supplementary information**

*Plasmodium* parasite malate-quinone oxidoreductase functionally complements a yeast deletion mutant of mitochondrial malate dehydrogenase

Takeshi Ito[1,2*], Sayaka Kajita[1,3], Minori Fujii[1,3], and Yasuo Shinohara[1,2]

[1]Institute of Advanced Medical Sciences, Tokushima University

[2]Graduate School of Pharmaceutical Sciences, Tokushima University

[3]Faculty of Pharmaceutical Sciences, Tokushima University

Running Head: *Plasmodium* MQO functions in place of mitochondrial MDH

[*]Correspondence: itou.takeshi@tokushima-u.ac.jp

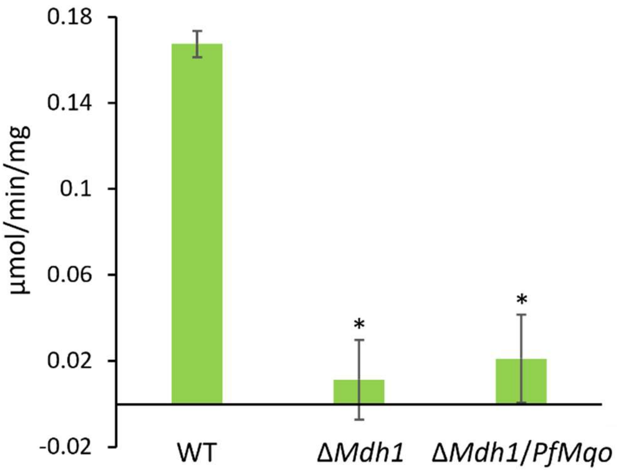


**Figure S1. Measurements of malate dehydrogenase activity in yeast mitochondrial fractions.** Mitochondrial fractions were isolated from yeast cells (WT, Δ*Mdh1* and Δ*Mdh1*/*PfMqo*) grown in CMLE medium. The malate dehydrogenase activity was measured using 5 mM malate and 200 μM β-$NAD^+$ as substrates, and malate-dependent reduction of β-$NAD^+$ was monitored. Measurements were repeated at least three times and the error bars show standard deviation of the mean. Data were analyzed by Dunnett's test (*, $P < 0.05$) comparing WT and each other sample. EZR software was used (1).

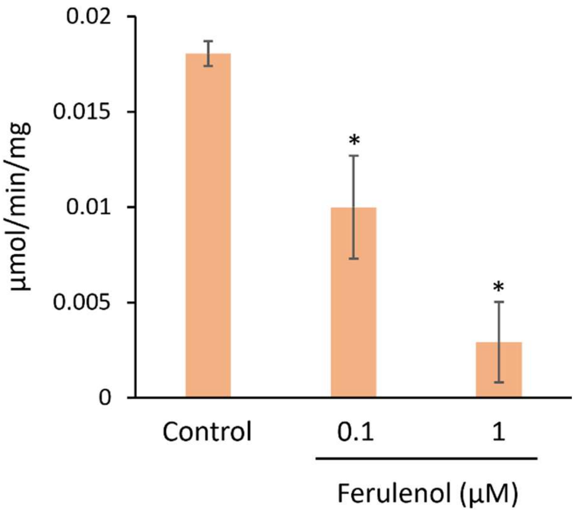


**Figure S2. The inhibitory effect of ferulenol on yeast succinate dehydrogenase.** A mitochondrial fraction was isolated from WT yeast cells grown in CMLE medium. The succinate dehydrogenase activity was measured using 60 μM decylubiquinone (dUQ) and 5 mM succinate as substrates, and dUQ-dependent reduction of DCIP was monitored. Where indicated, 0.1 or 1 μM ferulenol was added as an inhibitor. Measurements were repeated at least three times and the error bars show standard deviation of the mean. Data were analyzed by Dunnett's test (*, $P < 0.05$) comparing the control (no ferulenol) and each other sample. EZR software was used (1).

## Table S1. List of primers.

| Purpose | | Sequence 5' to 3' |
|---|---|---|
| Amplification of *Mdh1* deletion cassette | Forward | AAAAAAAACAAAAGGAAAAGGAAGGATACCATATACAATGtacgctgcaggtcgacaac |
| | Reverse | TTCCCTATTTTTCACTCTATTTCTGATCTTGAACAATCTAtaagggttctcgagagctcg |
| Amplification of *Mae1* deletion cassette | Forward | TTTTTTTTTTTAAGTGCAGGCGTTGGTTATGCTTCGTCTAgcctcgttcagaatgacacg |
| | Reverse | GCACATAAATACCAAGACAAAAGGTAGAAATACGGTTATGtgagcgctaggagtcactg |
| Amplification of *Dic1* deletion cassette | Forward | GCTATGTATCTTTATGTTTATATGTATATAAATCTGCCTAgtgcaccatatcgactacg |
| | Reverse | TGAAAGTGTCGAAAAGATAACGCAACAGCTGGACGGCATGaattggttgtttggccgagc |
| Verification of *Mdh1* deletion | Forward | CGTGGACATCTACGGAAAGG |
| | Reverse | TGGCGAGTAGTCTTCCGTTC |
| Verification of *Mae1* deletion | Forward | AAGTGCAGGCGTTGGTTATG |
| | Reverse | CCGTTACCGGCATGATTGAC |
| Verification of *Dic1* deletion | Forward | TACCTACCAACCGTGTAC |
| | Reverse | CTCTTGCTCTGAAAGTGTC |
| Addition of Flag tag into *PfMqo* | Forward | TACAAGTCAAAGGAGCCCC |
| | Reverse | aaaaggatcctacttgtcgtcatcgtctttgtagtcTAAGTAGTTTACAGGGTATTC |

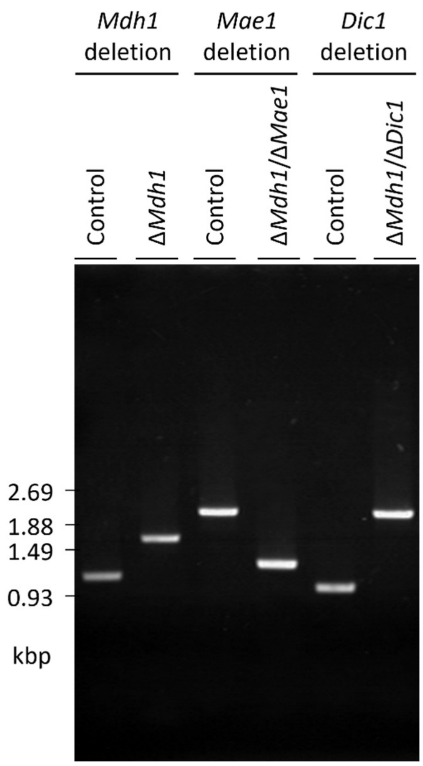


**Figure S3. Verification of gene deletions.** Genomic DNA was extracted from the transformants of Δ*Mdh1*, Δ*Mae1* and Δ*Dic1*. Gene deletions were verified by PCR using the genomic DNA preparations and external primers homologous to the flanking regions of the *Mdh1*, *Mae1* or *Dic1* genes (**Table S1**). The amplification products were analyzed on ethidium bromide-stained 1% agarose gels. Genomic DNA from each parental strain was used as a negative control. Amplification of the *Mdh1*, *Mae1* or *Dic1* genes with their flanking regions yields fragments of 1147 bp (WT) and 1679 bp (Δ*Mdh1*), 2197 bp (Δ*Mdh1*) and 1299 bp (Δ*Mdh1*/Δ*Mae1*), and 1027 bp (Δ*Mdh1*) and 2195 bp (Δ*Mdh1*/Δ*Dic1*), respectively.

**Materials and Methods**

*Malate dehydrogenase activity*: Mitochondrial fractions were isolated from WT, Δ*Mdh1* and Δ*Mdh1*/*PfMqo* strains grown in CMLE. The malate-dependent β-$NAD^+$ reduction in the mitochondrial fraction was measured spectrophotometrically at 25°C in a quartz cuvette. The absorbance change at 340 nm (ε = 6.22 $mM^{-1}$ $cm^{-1}$) was monitored, which shows the reduction of β-$NAD^+$. Mitochondrial fractions (0.05 mg/mL) were added to the assay buffer (1.8 mL), which contained 25 mM KPi pH 7, 0.65 M D-sorbitol, 5 mM $MgCl_2$, 2 μM antimycin A, and were incubated for 1 min, after which 5 mM L-malate was added. After an additional incubation for 1 min, the reaction was initiated by adding 200 μM β-$NAD^+$ (Sigma-Aldrich). Activity measurements were repeated at least three times.

*Succinate dehydrogenase activity*: Mitochondrial fractions were isolated from WT yeast grown in CMLE. The succinate-dependent ubiquinone reduction in the mitochondrial fraction was measured spectrophotometrically at 25°C in a quartz cuvette. DCIP was used as an indicator to detect the production of ubiquinol. The absorbance change at 600 nm (ε = 21 $mM^{-1}$ $cm^{-1}$) was monitored, which shows the reduction of DCIP by the ubiquinol produced. A portion of the mitochondrial fraction (0.05 mg/mL) and 120 μM DCIP (Sigma-Aldrich) were added to the assay buffer (1.8 mL) containing 25 mM KPi pH 7, 0.65 M D-sorbitol, 5 mM $MgCl_2$, 2 μM antimycin A, and were incubated for 1 min, after which 60 μM decylubiquinone (dUQ; Enzo Life Sciences) was added. After an additional incubation for 1 min, the reaction was initiated by adding 5 mM succinate. Where appropriate, 0.1 or 1 μM ferulenol (Adipogen Life Sciences) was added to the assay buffer. Activity measurements

were repeated at least three times.

## Reference

1. Kanda Y. 2013. Investigation of the freely available easy-to-use software 'EZR' for medical statistics. Bone Marrow Transplant 48:452-8.
